# Field theory model of brain extracellular matrix

**DOI:** 10.1093/jrr/rrt184

**Published:** 2014-03

**Authors:** Alexander Molochkov, Vladimir Goy, Anton Tolstonogov

**Affiliations:** School of Biomedicine, Far Eastern Federal University, Sukhanova 8, 690950 Vladivostok, Russia

**Keywords:** brain extracellular matrix, perineural net, lattice field theory, Ising model, brain signal processing; fractal structure

## Abstract

The perineural net (PNN) is responsible for synaptic stabilization of adult brain. It plays an important role in brain signal processing and non-synaptic signal transfer as well [ 1]. Since it is composed of largely negatively charged chains of disaccharides, it can be easily affected by strong external electromagnetic field irradiated by high-energy particles passing brain tissues. One of the effects of such exposure is a cognitive impairment. Since outside of the Bragg peak area local electromagnetic field irradiation strong enough to affect on the PNN's dynamics, it is very important to understand PNN's behavior in locally strong electromagnetic fields. The presented work is devoted to PNN modeling in field theory framework. As a toy model of PNN, we consider fractal lattice with Ising and XY model applied. More precise study requires application of the quantum electrodynamics on the lattice. We study how local excitations can change phase state of the overall lattice and its finite clusters. For this propose, the partition function of the Ising model is calculated using Monte Carlo simulation [ 2]. The two-dimensional Serpinsky Carpet lattice is applied to approximate structure of the brain extracellular matrix. Order parameter is calculated for different levels of the self-similarity of the Serpinsky Carpet. It is shown that starting from the level = 3, the lattice with fractal structure has irreversible phase transition: phase state after restoration differs from the initial one. In Fig. 1, the calculation of the correlation length change due to the energy dissipation after a local impact is shown. The column shows correlation strength (from 0 to 1), the right axis shows the cluster size ratio to the whole lattice size in percentage. The initial lattice was totally correlated (100% of the lattice has correlation strength that equals 1). The left side of the picture corresponds the locally excited lattice with no correlation, the right side of the picture corresponds the lattice after the relaxation to the equilibrium state due to the energy dissipation. It can be clearly seen that the lattice is not correlated after the relaxation.

**Conclusions:**

Local impact changes the correlation length of the fractal lattice: after the restoration, the correlation length becomes short, thus signal processing on the lattice changes significantly. Thus, it can be considered as a good model to study of the extracellular matrix change due to local excitations of strong electromagnetic field.

**Fig. 1.**
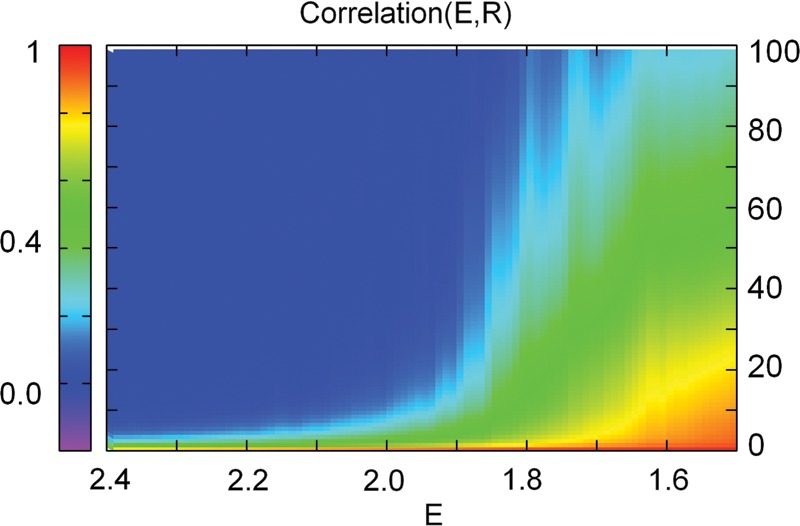
Correlation length change due to the energy dissipation.
